# Towards Best Practice in Hair Metabolomic Studies: Systematic Investigation on the Impact of Hair Length and Color

**DOI:** 10.3390/metabo10100381

**Published:** 2020-09-26

**Authors:** Lisa Eisenbeiss, Tina M. Binz, Markus R. Baumgartner, Thomas Kraemer, Andrea E. Steuer

**Affiliations:** 1 Department of Forensic Pharmacology & Toxicology, Zurich Institute of Forensic Medicine, University of Zurich, 8057 Zurich, Switzerland; lisa.eisenbeiss@irm.uzh.ch (L.E.); thomas.kraemer@irm.uzh.ch (T.K.); 2 Center for Forensic Hair Analytics, Zurich Institute of Forensic Medicine, University of Zurich, 8006 Zurich, Switzerland; TinaMaria.Binz@irm.uzh.ch (T.M.B.); markus.baumgartner@irm.uzh.ch (M.R.B.)

**Keywords:** LC-MS/MS, hair metabolomics, hair analysis, endogenous compounds, segmentation, hair color

## Abstract

Untargeted metabolomic studies are used for large-scale analysis of endogenous compounds. Due to exceptional long detection windows of incorporated substances in hair, analysis of hair samples for retrospective monitoring of metabolome changes has recently been introduced. However, information on the general behavior of metabolites in hair samples is scarce, hampering correct data interpretation so far. The presented study aimed to investigate endogenous metabolites depending on hair color and along the hair strand and to propose recommendations for best practice in hair metabolomic studies. A metabolite selection was analyzed using untargeted data acquisition in genuine hair samples from different hair colors and after segmentation in 3 cm segments. Significant differences in metabolites among hair colors and segments were found. In conclusion, consideration of hair color and hair segments is necessary for hair metabolomic studies and, subsequently, recommendations for best practice in hair metabolomic studies were proposed.

## 1. Introduction

In recent years, metabolomic studies have gained significant importance. As the end of the ‘omics’ cascade, metabolomics directly reflects modified metabolic pathways provoked by systems disruption as a consequence of, e.g., diseases or exogenous causes [1]. This permits a deeper understanding of metabolic pathways in general and in particular a biomarker search for specific questions. As a holistic approach, untargeted analysis of metabolites enables an objective study of the metabolome by monitoring metabolomic changes without prior restriction to a selection of metabolites [2].

To date, metabolomic studies have mostly been performed in blood or urine samples. As sample handling and data interpretation have been well studied, comparability and reliability of analysis results are a given. However, dynamic fluctuations in the metabolome (such as dietary and circadian variations, lifestyle habits or immune status) can significantly affect the metabolome. Thus, small or short-term changes of the metabolome as a consequence of a monitored effect might go unnoticed. To that end, hair was introduced as a new matrix for metabolomic studies for long-term and retrospective analysis of metabolome changes [3,4,5,6]. Hair metabolomics is based on the fact that endogenous metabolites are constantly incorporated into the growing hair shaft and that small molecules are conserved within the stable keratinized hair structure. The constant accumulation of endo- and exogenous compounds might allow the search for robust biomarkers omitting short-term fluctuations. Additionally, with an average hair growth rate of 1 cm/month, segmentation of hair samples theoretically allows for retrospective and chronological tracking of metabolite changes [7]. Its non-invasive sampling and easy storage furthermore favors its application for metabolomic studies.

Common models propose the incorporation of exogenous compounds via blood and sweat or sebum. The incorporation itself depends on different compound-specific physico-chemical properties such as pK_a_, molecular weight, lipophilicity or basicity. In addition to these factors, incorporation into the hair shaft is also influenced by the melanin content of hair (the so-called ‘melanin effect’) [7]. In contrast to blood or urine samples, scalp hair samples are constantly exposed to the environment, which affects the hair matrix. Sunlight and UV exposure, regular hair washing and cosmetic hair treatments affect the integrity of the hair structure, leading to altered detectability of exogenous analytes in hair with increasing age of the hair samples [7,8]. On that basis, likewise incorporation and behavior of endogenous compounds is assumed. This would imply that endogenous compounds might be substantially affected through different mechanisms simply because of a hair sample’s nature and relative concentration changes as monitored in untargeted metabolomic studies might be misinterpreted. To that end, interpretation and comparability of hair metabolomic results would significantly depend on a standardized pre-analytical workflow as well as on the hair sample itself.

In general, due to its dynamic nature, metabolomic studies should be standardized as much as possible to ensure data reliability and consistent communication of results for better comparability. So far, efforts in this direction have been made from the metabolomics standards initiative (MSI) [9] for minimum reporting standards for metabolomics data or by publications aiming to harmonize/standardize the method development for common untargeted metabolomic studies [10,11]. However, these studies usually cover only the handling of classical matrices and fundamental data for hair metabolomics are rare. So far, only one study systematically investigated standardization methods specifically for hair metabolomics [12]. However, information on the behavior of endogenous compounds simultaneously covering different compound classes, as is the case in untargeted metabolomic studies, is scarce but indispensable for reliable data interpretation.

Within the present study, the aim was to systematically investigate the general behavior of a selection of endogenous metabolites from different compound classes in genuine hair samples in order to highlight general pitfalls in hair metabolomics and improve future hair metabolomic study designs and data interpretation. For this, the influence of the hair length and the hair color on the detectability of endogenous compounds was investigated.

## 2. Results and Discussion

### 2.1. Hair Color Dependencies of Endogenous Compounds: Interindividual Differences

**Figure 1:**
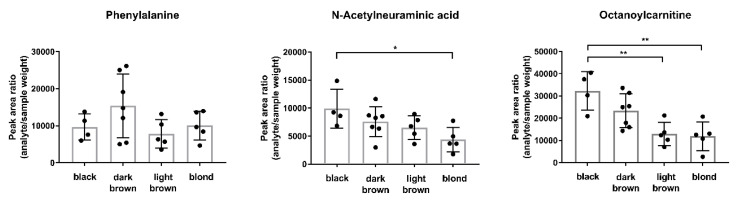
Distribution of endogenous compounds over different hair colors. Depicted are analyte peak area ratios (mean and SD) (black n = 4, dark–medium brown n = 7, light brown–dark blond n = 5, and blond n = 5) for three exemplary analytes. Statistical analysis with ordinary one-way ANOVA. *p* > 0.05 (not significant, without indication); *p* ≤ 0.05 (*); *p* < 0.01 (**).

Metabolites chosen for this study have been selected from metabolites previously encountered and described in hair metabolomics studies [6,12]. Focus was applied to include a number of metabolites that were both from various chemical classes and unambiguously identifiable (i.e., a reference substance was available). Exemplified hair color dependencies and non-dependencies observed in the current data set from black to light blond are shown in Figure 1. Summarized results of hair color dependencies for all investigated metabolites can be found in Table 1. Twenty percent of the metabolites differed significantly between hair colors. We mainly observed differences between melanin-rich black hair samples and melanin-poor light blond hair samples, implying a possible hair color-dependent incorporation preference for some endogenously present metabolites. It is known that, in addition to the physico-chemical properties of metabolites themselves, hair pigmentation can substantially influence incorporation of drugs into the hair shaft (‘melanin effect’). On the one hand, melanin shows a significant affinity for basic drugs. On the other hand, pH values between 3 and 5 within melanocytes provoke protonation of basic drugs once they pass the membrane barrier. These effects have been shown to lead to an accumulation and favored incorporation of basic drugs into pigmented hair samples [7]. Likewise, melanin/hair color-dependent incorporation of endogenous compounds in the current study is most likely. For instance, in our data set, no hair color dependency was observed for the relatively neutral amino acid phenylalanine, whereas hair color dependency was increasingly observed for *N*-acetylneuraminic acid and octanoylcarnitine, with increasing pK_a_ values respectively (see Figure 1). As melanin affinity is favored for basic drugs, a correlation of significant hair color dependencies and pK_a_ values was, subsequently, attempted. However, a prediction of the behavior pattern of single metabolites or whole compound classes was not yet possible. To that end, except for cysteic acid, amino acids did not show significant hair color dependencies, whereas other compound classes showed varying patterns (see Table 1). For instance, increasing hair color dependency was observed for carnitines with increasing chain length. Hence, in addition, lipophilicity might eventually contribute to hair color-dependent incorporation of endogenous analytes. Furthermore, eumelanin was previously shown to act as a photoprotective agent in hair samples [13]. Lower melanin concentrations in light or non-pigmented hair samples might therefore promote enhanced photodegradation of other endogenous metabolites compared to black hair samples, possibly pretending metabolome changes.

In conclusion, concentrations of endogenous metabolites can vary significantly among different hair colors. Particularly when analyzing a whole set of metabolites, one should be aware that some metabolites are more strongly affected by hair color dependencies than others. In metabolomic studies in general, unwanted sources of variation should be reduced and hence incorporation effects should be excluded as much as possible. Hair colors should therefore be considered at the time of the study design and at the latest during data interpretation to avoid any false-positive findings and misinterpretations associated with the hair color. We therefore recommend a balanced distribution of different hair colors within the sample cohorts and between control and effect groups. At best, hair samples from one hair color only should be included. If this cannot be ensured, hair colors should be reported and analysis results interpreted with care.

### 2.2. Segmental Hair Analysis of Cosmetically Untreated Hair Samples: Intra-Individual Differences

**Figure 2:**
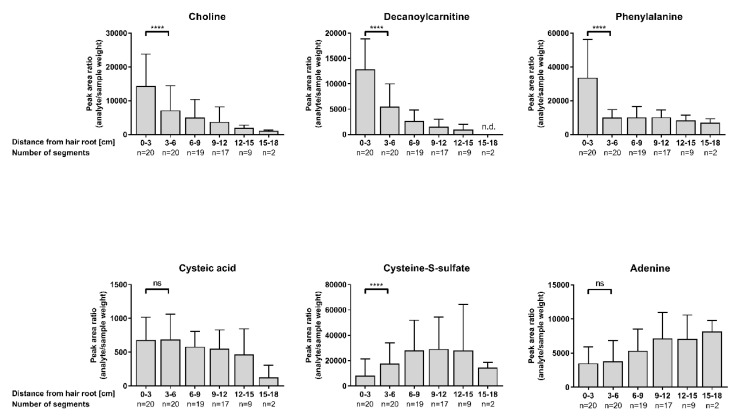
Depicted are the mean and standard deviations of analyte peak area ratios (peak area analyte/sample weight) of a selection of metabolites in cosmetically untreated hair samples, segmented into 3 cm segments starting from the root end of the hair shaft. n.d.: not detected. Statistical analysis between first and second segment with Wilcoxon matched pairs signed rank test; ns: not significant; *p* < 0.0001 (****).

Due to the stable character of the keratinized matrix, one of the main advantages of hair samples compared to other matrices is its exceptional long detection window for incorporated substances. The detection window is defined by the hair growth rate and growth cycle. Generally, an average growth rate of 1 cm/month can be assumed which permits the retrospective and time-resolved analysis of (drug) consumption history and, eventually, metabolomic changes up to years. However, this long detection window also presents the biggest challenge for hair metabolomics, as controlled studies over such a long time period are almost impossible. Therefore, general knowledge of the behavior of endogenous compounds along the hair strand is crucial if retrospective metabolome changes are to be evaluated. To that end, hair samples were analyzed in 3 cm segments. To exclude hair color effects as much as possible, black and light blond hair samples were excluded from the study (details of the hair samples used can be found in Table 2). Segmentation results for a selection of metabolites are depicted in Figure 2. Detailed results for all metabolites are summarized in Table 1. Except for cysteic acid, clear differences between hair segments could be observed for all metabolites throughout the different compound classes. Highest signals for metabolites were observed for the proximal 3 cm segment with only few exceptions. Signals continually decreased from proximal to distal for the majority of metabolites, as illustrated for choline and decanoylcarnitine in Figure 2; other metabolites such as phenylalanine showed a significant decrease after the first segment but stayed unchanged thereafter. Only cysteine-sulfate and adenine showed an increase towards distal segments. These results are in line with studies from Noppe et al., who found a gradual decrease in steroid concentrations from proximal towards distal ends after segmentation in 3 cm segments. Additionally, the authors divided the first proximal 3 cm segment into 1 cm segments and could already observe a concentration decrease within the first 3 cm of the hair shaft (proximal to distal), which implies that this could be observable for other endogenous compounds if narrower segments are chosen [14].

Decreasing signals of metabolites towards distal hair segments are not surprising, as hair samples are constantly exposed to environmental factors such as regular hair washing and UV exposure (so called weathering or aging effects). In particular, solar radiation is known to cause hair damage by direct interaction with hair components or through the formation of reactive oxygen species (ROS) mainly through the reaction of molecular oxygen (O_2_) with melanin radicals [15]. For instance, in vitro studies confirmed that (UV-)light exposure leads to decreased concentrations of cortisol and cortisone in hair samples [16]. Accordingly, cysteine-sulfate, an oxidation product of cystine recently found to be formed after oxidation [6], increased towards distal segments. Furthermore, increased damage of the hair structure through photodamage might make metabolites even more susceptible to enhanced wash-out effects over time. As a consequence, a detailed questionnaire on sunlight exposure, solarium visits and holiday habits as well as the sampling time point (summer or winter) should be considered and documented especially when segmented hair analysis is planned. For this, we refer to a questionnaire by Grass et al. about the estimation of sunlight exposure [16]. In our study, hair samples from ten individuals were sampled within 6 months distance in spring and in fall, reflecting either summer or winter months (see Table 1). Metabolite signals in the respective proximal 3 cm segment were compared and evaluated for a potential difference. Assuming enhanced degradation through sunlight and expected higher sunlight exposition times during summer months, lower signals for most analytes in hair specimens sampled in fall were expected. This could be significantly observed for single metabolites (carnitine, hexanoylcarnitine, butyrylcarnitine, glutamic acid and indole-3-lactic acid) and a tendency towards increased signals in fall samples for cysteine-sulfate could be detected accordingly.

Recently, a study by Delplancke et al. investigated the longitudinal metabolite profile in segmented hair samples from healthy pregnant women corresponding to the three trimesters of pregnancy [17]. They identified significantly altered metabolites between the different trimesters. However, these changes were solely linked to the different trimesters of the pregnancy but no corresponding hair segments from a control group were investigated for comparison. Considering the significant changes observed between the first and second segments in our study, these results might need re-evaluation to account for hair length/segment variations. In general, this seems to be a substantial issue in current hair metabolomic studies, as the information on hair lengths are, if at all, poorly reported. This highlights the urgent need for standardized protocols for hair metabolomic studies.

In general, in addition to exogenous hair aging, commonly known factors such as sweat contamination, dietary or lifestyle habits that may significantly affect the detected metabolic profile along hair samples need to be considered. Additionally, analytical variations also contribute to the obtained metabolic profile, particularly sample preparation and extraction variabilities [12]. That is why a suitable sample size should be a prerequisite for conclusive statements from a hair metabolomics study. Furthermore, we recommend that comparison of analysis results between individuals should only be performed within same hair segments to avoid incorrect findings. As a result, for retrospective intra-individual metabolome profiling, more frequent hair sampling and analysis of the same hair segments is recommended to cover a longer time period rather than a single sample collection which comes with elongated segmentation steps. Further, in regard to impaired time resolution with increasing hair length [7], this effort should be taken to avoid wash-out effects as much as possible. Hence, suitable control groups and a thorough study design are indispensable.

### 2.3. General Recommendations for Best Practice in Hair Metabolomics Studies and Future Perspectives

Within this study, we could show that the hair metabolome depends on the hair color and changes along the hair strand in an entirely natural way. Additionally, it has been shown that cosmetic hair treatments significantly affect the detectability of endogenous compounds [6,18,19]. These aspects are not considered in published hair metabolomic studies so far, which highlights the urgent need to raise attention to this topic. In general, metabolomic studies should follow standardized procedures to assure proper data quality and confidently relate metabolome changes to an observable effect. Recommendations or guidelines for the conduction of hair metabolomic studies, as already established in forensics and workplace drug testing [20,21], are still missing to date. As such, we propose some minimum reporting standards for best practice procedures in hair metabolomic studies and give recommendations for the study design of future hair metabolomic studies.

For hair metabolomic study design, a suitable sample size is recommended and a thorough questionnaire to estimate influences on the hair matrix is indispensable. This questionnaire should include the following documentation: hair color, any information on cosmetic and other hair treatments, hair washing habits and documentation on (UV-)light exposures. Information specifically important for metabolomic studies such as lifestyle habits (e.g., smoking behavior), drug intake, sport activities or state of health should be included depending on the question of interest. In addition to the common reporting standards proposed by the MSI [9], metabolome studies in hair should report the analyzed hair segments or hair lengths, and possibly present stubble lengths, hair color and cosmetic treatment.

Even though hair metabolomics is still in its infancy, with a reasonable study design and appropriate data interpretation, it can be an alternative when other matrices reach their limitations due to shorter preservation of metabolome changes and their high dynamic nature.

### 2.4. Limitations of the Study

Our study had some limitations. Only a small selection of a high diversity of metabolites that represent the global metabolome was analyzed. However, the compounds targeted covered the currently known metabolite classes detectable in hair samples. As not every compound class could be included in the study design, some tendencies and metabolite behaviors might be missing to fully explain and understand hair color-dependent detection of metabolites. Further, the sample size was too small for full elucidation of the underlying mechanisms involved in hair color-dependent incorporation. However, to indicate the relevance of differences between hair color and hair length, the sample size was suitable and fully supports the key messages of our findings.

## 3. Materials and Methods

### 3.1. Chemicals and Reagents

Analytical reference standards of all metabolites investigated within this study were purchased from Sigma-Aldrich (Buchs, Switzerland). All internal standards (IS) were purchased from Cambridge Isotope Laboratories, Inc (Andover, MA, USA) and either delivered by ReseaChem Life Science (Burgdorf, Switzerland) or Sigma-Aldrich (Buchs, Switzerland). HPLC-grade methanol (MeOH), dichloromethane (DCM), acetonitrile (ACN) and ultrapure water were sourced from Fluka (Buchs, Switzerland). All other chemicals used were from Merck (Zug, Switzerland) and of the highest grade available.

### 3.2. Hair Samples

Genuine hair samples were collected and anonymized from routine case work from the Center for Forensic Hair Analytics (Zurich, Switzerland) and from volunteers who provided written informed consent. All samples were analyzed in full conformance with Swiss laws (statement of Cantonal Ethics Board of the Canton of Zurich: BASEC-Nr. Req-2017-00946). According to Swissethics (Humanforschungsgesetz), no further ethical approval from the cantonal ethic commission is necessary if the research is not aiming to investigate diseases or functions of the human body as is the case in the current study. All hair samples were collected with a pair of scissors at the vertex posterior region close to the scalp (0.3–0.5 cm) and stored in aluminum foil at room temperature. Samples were categorized in two cohorts: cohort 1 (segmentation of hair strands in 3 cm segments; n = 20) and cohort 2 (analysis of potential hair color dependencies of endogenous analytes, analysis of the proximal 3 cm segments; black: n = 4, dark brown–medium brown: n = 7, light brown–dark blond: n = 5 and medium blond–light blond: n = 5). Detailed information on hair samples from cohort 1 can be found in Table 2.

### 3.3. Hair Sample Preparation

Hair sample preparation was performed according to Eisenbeiss et al. [6]. In brief, hair decontamination was conducted by successive washing with dichloromethane (DCM), acetone, H_2_O and acetone. A total of 20–30 mg hair was pulverized and extraction was performed with 1 mL ACN/H_2_O (2:8, *v/v*) and 20 µL IS solution (adenosine ribose-D_1_ (0.075 mM), arginine-^13^C_6_ (1.5 mM), caffeine 3-methyl-^13^C (1 mM), carnitine trimethyl-D_9_ (0.5 mM), deoxycholic acid-D_4_ (0.009 mM), D-fructose ^13^C (0.6 mM), hippuric acid ^15^N (2.5 mM), leucine-D_10_ (1.5 mM), lysine-D_4_ (3.5 mM), phenylalanine-D_1_ (1.5 mM), proline ^15^N (3.5 mM) and tryptophan-D_5_ (1.25 mM)) during ultra-sonication over 16 h. After centrifugation (5 min, 9000 rpm), the supernatant was evaporated under nitrogen and reconstituted with 250 µL ACN/H_2_O (2:8, *v/v*). Finally, reconstituted extracts were purified by centrifugation and filtration (5 min, 9000 rpm, VWR centrifugal filter, 0.45 µm pore size, VWR, Dietikon, Switzerland). To assure the quality of analysis results, a solvent blank was prepared in exactly the same way as the study samples (correction of false-positive results) and pooled QC samples were obtained by mixing 30 µL of each hair extract together.

### 3.4. Liquid Chromatography High-Resolution Mass Spectrometry (HPLC-HRMS)

The study data were obtained by untargeted data acquisition methods using LC-HRMS with two chromatographic columns in the positive and negative ionization modes as commonly applied in untargeted metabolomic studies [2]. All samples were analyzed as described in previously published studies [6,22] and in randomized order on a Thermo Fischer Ultimate 3000 UHPLC system (Thermo Fischer Scientific, San Jose, CA), coupled to a high-resolution (HR) time-of-flight (TOF) instrument system (TripleTOF 6600, Sciex, Concord, ON, Canada). Methodological details and performance evaluation have been described in detail elsewhere [22]. In brief, chromatographic separation was achieved by using reversed-phase (Xselect HSST RP-C18 (150 mm × 2.1 mm i.d.; 2.5 µm particle size) from Waters (Baden-Daettwil, Switzerland)) and hydrophilic interaction liquid chromatography (HILIC) (Merck (SeQuant ZIC HILIC column, 150 mm × 2.1 mm, 3.5 µm particle size)) with gradient elution (mobile phase A: 10 mM ammonium formate in water containing 0.1% (*v/v*) formic acid; mobile phase B: MeOH containing 0.1% (*v/v*) formic acid; mobile phase C: 25 mM ammonium acetate containing 0.1% (*v/v*) acetic acid in water; and mobile phase D: ACN containing 0.1% (*v/v*) acetic acid). For all samples, 5 µL were injected. HR mass spectra (MS) and tandem mass spectrometry (MS/MS) data were acquired in TOF-MS only and information-dependent acquisition (IDA) in the positive and negative electrospray ionization (ESI) modes. The MS analysis was performed with a DuoSpray ion source at a resolving power (full width at half maximum (fwhm) at *m*/*z* 400) of 30,000 in MS and 30,000 in MS2 (the HR mode) or 15,000 (the high-sensitivity mode). For every 5th sample in the positive and every 3rd sample in the negative ionization mode, automatic calibration was performed using an atmospheric-pressure chemical ionization (APCI) positive or negative calibration solution (Sciex, Concord, ON, Canada), respectively. TOF-MS criteria were set as follows: mass range *m*/*z* 50 to 1000, accumulation time 100 msec, and collision energy (CE) 5 eV. IDA experiments were performed with the following settings: mass range *m*/*z* 50 to 1000, accumulation time 100 ms, CE 35 eV with CE spread 15 eV, dynamic background subtraction, 4 most intense ions, intensity threshold above 100 cps and exclusion time of 5 s (half peak width) after two occurrences. MS data acquisition was controlled by Analyst TF software 1.7 (Sciex, Concord, ON, Canada).

For column equilibration, a series of QC Pools was injected at the sequence beginning and then repeatedly after every 5th sample. A system suitability test (SST) was prepared according to previously published protocols [22,23] and was injected every 10th sample to check data reproducibility and correct for any retention time (RT) shifts. Targeted peak area integration of SST analytes and endogenous compounds was performed by peak integration of precursor ions from TOF-MS runs using Multiquant V 2.1 (Sciex, Concord, ON, Canada). In addition to retention times and accurate precursor masses, MS/MS spectra from IDA runs were used as further metabolite identification criteria only. Direct comparison of analysis results was performed only with samples from the same batch.

### 3.5. Data Processing and Statistics

Although targeted evaluation of a metabolite selection was conducted within this study, relative abundance changes of analyte peak areas were considered as if these samples would be processed within an untargeted hair metabolomics study. Analyte peak areas were obtained after integration of precursor ions ([M + H]^+^ or [M − H]^−^) of full-scan data and were normalized by the respective sample weight. Statistical analyses were performed in GraphPad Prism 7 (GraphPad Software, San Diego, CA, USA).

## 4. Conclusions

Within this study, we demonstrated and highlighted the challenges for hair metabolomic studies in general and when profiling the hair metabolome among different hair colors and along the hair strand. For reliable metabolomics data interpretation, a highly standardized and well-planned study design is crucial. So far, no recommendations for conducting hair metabolomic studies exist. As the detection of endogenous metabolites can depend significantly on the hair color and the analyzed hair segments, we recommend reporting these parameters in future hair metabolomic studies to ensure transparency for minimum data quality of the results. With these new findings, a step towards the successful implementation and conduction of hair metabolomic studies was achieved. Further systematic studies are needed to extend the knowledge on the hair metabolome, which will eventually contribute to higher quality standards in hair metabolomic studies.

## Figures and Tables

**Table 1:** Summarized are analyzed metabolites and their behavior depending on hair color (cohort 2) and after hair segmentation (cohort 1). Statistical analysis for hair color dependencies with ordinary one-way ANOVA (with application of Tukey’s test for multiple comparison) and application of Wilcoxon matched pairs rank test for statistical analysis between hair segments. *p* > 0.05: not significant (n.s.); *p* ≤ 0.05 (*); *p* < 0.01 (**); *p* < 0.001 (***); *p* < 0.0001 (****). Hair segments: A (0–3 cm), B (3–6 cm), C (6–9 cm) and D (9–12 cm).

Analyte	Compound Class	Formula	pK_a_	Analytics	Differences between Hair Colors	Differences between Hair Segments					
						A vs. B	B vs. C	A vs. C	A vs. D	B vs. D	C vs. D
**Choline**	1,2-Aminoalcohols	C_5_H_14_NO	13.97	HSST ESI ^+^	black to blond: ↓ *	↓ ****	↓ *	↓ ****	↓ ****	↓ **	↓ **
**Cysteic acid**	Amino acids and derivatives	C_3_H_7_NO_5_S	−1.7	HILIC ESI ^−^	black to blond: ↓ **	n.s.	n.s.	n.s.	n.s.	n.s.	n.s.
					black to light brown: ↓ *						
**Urocanic acid**	Amino acids and derivatives	C_6_H_6_N_2_O_2_	3.85	HILIC ESI ^+^	n.s.	↓ ****	↓ *	↓ ****	↓ ***	n.s.	n.s.
**Citrulline**	Amino acids and derivatives	C_6_H_13_N_3_O_3_	2.27	HILIC ESI ^−^	n.s.	↓ ****	↓ ***	↓ ****	↓ ****	↓ ***	↓ **
**Trimethyllysine**	Amino acids and derivatives	C_9_H_20_N_2_O_2_	2.41	HSST ESI ^+^	n.s.	↓ ****	↓ *	↓ ****	↓ ****	↓ *	↓ **
**Pyroglutamic acid**	Amino acids and derivatives	C_5_H_7_NO_3_	3.61	HILIC ESI ^−^	n.s.	↓ ****	↓ **	↓ ****	↓ ****	↓ ***	↓ *
**Cysteinesulfate**	Amino acids and derivatives	C_3_H_7_NO_5_S_2_	−1.9	HILIC ESI ^−^	n.s.	↑ ****	↑ ***	↑ ****	↑ ****	↑ ***	n.s.
**Ornithine**	Amino acids and derivatives	C_5_H_12_N_2_O_2_	2.67	HILIC ESI ^+^	n.s.	↓ ****	n.s.	↓ *	↓ *	n.s.	n.s.
**Indole-3-lactic acid**	Amino acids and derivatives	C_11_H_11_NO_3_	4.14	HSST ESI ^+^	n.s.	↓ ****	↓ **	↓ ***	↓ ***	↓ **	↓ ***
**Taurine**	Amino acids and derivatives	C_2_H_7_NO_3_S	−1.5	HILIC ESI ^-^	n.s.	↓ ***	↓ ***	↓ ****	↓ ****	↓ **	n.s.
**Creatinine**	Amino acids and derivatives	C_4_H_7_N_3_O	9.21	HSST ESI ^+^	n.s.	↓ ****	↓ *	↓ ****	↓ ****	↓ *	↓ *
**Serine**	Amino acids and derivatives	C_3_H_7_NO_3_	2.03	HILIC ESI ^−^	n.s.	↓ ****	↓ *	↓ ****	↓ ****	↓ *	n.s.
**Arginine**	Amino acids and derivatives	C_6_H_14_N_4_O_2_	2.41	HSST ESI ^+^	n.s.	↓ ****	n.s.	↓ ****	↓ ****	n.s.	↓ *
**Lysine**	Amino acids and derivatives	C_6_H_14_N_2_O_2_	2.74	HILIC ESI ^+^	n.s.	↓ ****	n.s.	↓ ****	↓ ****	n.s.	n.s.
**Phenylalanine**	Amino acids and derivatives	C_9_H_11_NO_2_	2.47	HSST ESI ^+^	n.s.	↓ ****	n.s.	↓ ****	↓ ****	n.s.	n.s.
**Tyrosine**	Amino acids and derivatives	C_9_H_11_NO_3_	2.0	HILIC ESI ^+^	n.s.	↓ ****	↓ *	↓ ****	↓ ****	n.s.	n.s.
**Tryptophan**	Amino acids and derivatives	C_11_H_12_N_2_O_2_	2.54	HSST ESI ^+^	n.s.	↓ ****	n.s.	↓ ****	↓ ****	n.s.	↓ *
**Glutamic acid**	Amino acids and derivatives	C_5_H_9_NO_4_	1.88	HSST ESI ^+^	n.s.	↓ ****	↓ **	↓ ****	↓ ****	↓ **	↓ **
**Aspartic acid**	Amino acids and derivatives	C_4_H_7_NO_4_	1.7	HSST ESI ^-^	n.s.	↓ ****	↓ *	↓ ****	↓ ****	↓ *	n.s.
**Histidine**	Amino acids and derivatives	C_6_H_9_N_3_O_2_	1.85	HSST ESI ^+^	n.s.	↓ *	n.s.	↓ *	↓ ***	n.s.	n.s.
**N-Acetylneuraminic acid**	Carbohydrates and carbohydrate conjugates	C_11_H_19_NO_9_	3.0	HILIC ESI ^+^	black to blond: ↓ *	↓ **	↓ *	↓ ****	↓ ****	↓ **	↓ **
**Carnitine (C0)**	Carnitines	C_7_H_15_NO_3_	4.2	HSST ESI ^+^	n.s.	↓ ****	↓ ****	↓ ****	↓ ****	↓ ****	↓ **
**Acetylcarnitine (C2)**	Carnitines	C_9_H_17_NO_4_	4.09	HILIC ESI ^+^	n.s.	↓ ****	↓ *	↓ ****	↓ ****	↓ **	↓ *
**Butyrylcarnitine (C4)**	Carnitines	C_11_H_21_NO_4_	4.27	HILIC ESI ^+^	n.s.	↓ ****	n.s.	↓ ****	↓ ****	↓ *	n.s.
**Hexanoylcarnitine (C6)**	Carnitines	C_13_H_25_NO_4_	4.22	HSST ESI ^+^	black to blond: ↓ *	↓ ****	↓ *	↓ ****	↓ ****	↓ *	n.s.
					black to light brown: ↓ *						
**Octanoylcarnitine (C8)**	Carnitines	C_15_H_29_NO_4_	4.22	HSST ESI ^+^	black to blond: ↓ **	↓ ***	↓ **	↓ ****	↓ ****	↓ ***	n.s.
					black to light brown: ↓ **						
**Decanoylcarnitine (C10)**	Carnitines	C_17_H_33_NO_4_	4.22	HSST ESI ^+^	black to light brown: ↓ *	↓ ****	↓ ***	↓ ****	↓ ****	↓ ****	↓ **
**Riboflavin**	Cofactors	C_17_H_20_N_4_O_6_	5.97	HSST ESI ^+^	black to blond: ↓ **	↓ ***	n.s.	↓ ***	↓ ***	↓ *	↓ *
**Nicotinamide**	Cofactors	C_6_H_6_N_2_O	13.39	HSST ESI ^+^	n.s.	↓ **	n.s.	↓ **	↓ ****	n.s.	n.s.
**Inosine**	Nucleosides and nucleotides	C_10_H_12_N_4_O_5_	6.94	HILIC ESI ^−^	n.s.	↓ **	n.s.	↓ ***	↓ ***	↓ **	↓ **
**Adenosine**	Nucleosides and nucleotides	C_10_H_13_N_5_O_4_	12.45	HSST ESI ^+^	n.s.	↓ **	n.s.	↓ ****	↓ ****	n.s.	n.s.
**Adenine**	Nucleosides and nucleotides	C_5_H_5_N_5_	10.29	HSST ESI ^+^	n.s.	n.s.	↑ *	n.s.	↑ ***	↑ ***	↑ *
**Theobromine**	Purines and derivatives	C_7_H_8_N_4_O_2_	9.28	HSST ESI ^+^	n.s.	↓ ****	↓ **	↓ ****	↓ ****	↓ **	↓ ***
**Theophylline**	Purines and derivatives	C_7_H_8_N_4_O_2_	7.82	HILIC ESI ^+^	black to blond: ↓ *	↓ ***	↑ **	↓ ***	↓ ****	↓ **	↓ *
					black to light brown: ↓ *						
					black to dark brown: ↓ *						
**Hypoxanthine**	Purines and derivatives	C_5_H_4_N_4_O	8.72	HSST ESI ^+^	n.s.	↓ ***	↓ **	↓ ****	↓ ****	↓ *	↓ *
**Uric acid**	Purines and derivatives	C_5_H_4_N_4_O_3_	7.25	HILIC ESI ^−^	n.s.	↓ **	n.s.	↓ ***	↓ ****	↓ **	↓ **

↓: mean concentration decrease, ↑: mean concentration increase; “+”: positive ionization mode, “-“: negative ionization mode.

**Table 2:** Detailed hair sample characteristics of hair samples used for segmentation (cohort 1).

Subject Number	Hair Color	Sampling Month	Hair Length (cm)	Number of Analyzed Segments
**1**	dark blond	05/2019	15	5
		11/2019	15	4
**2**	dark blond	05/2019	16	5
		11/2019	16	4
**3**	dark blond	05/2019	14	5
		11/2019	18	4
**4**	dark brown	11/2018	35	4
		05/2019	32	4
**5**	dark brown	11/2018	37	4
		05/2019	12	3
**6**	dark brown	11/2018	24	5
		05/2019	33	5
**7**	dark brown	11/2018	12	4
		05/2019	8	2
**8**	dark brown	10/2018	16	5
		04/2019	14	3
**9**	medium brown	05/2019	23	6
		11/2019	25	6
**10**	medium brown	10/2018	28	4
		04/2019	25	5
